# Open Fractures Beyond Long Bones: Rethinking Age-Dependent Injury Mechanisms and Anatomical Patterns in Pediatric Trauma

**DOI:** 10.3390/children13070918

**Published:** 2026-07-12

**Authors:** Britta Chocholka, Lara Marie Bogensperger, Vanessa Groß, Antonia Schwarz, Stephan Payr, Manuela Jaindl

**Affiliations:** 1 University Clinic of Orthopedics and Trauma Surgery, Department of Trauma Surgery, Medical University of Vienna, Waehringer Guertel 18-20, 1090 Vienna, Austria; n11807478@students.meduniwien.ac.at (L.M.B.); n11916964@students.meduniwien.ac.at (V.G.); n12220006@students.meduniwien.ac.at (A.S.); stephan.payr@meduniwien.ac.at (S.P.); manuela.jaindl@meduniwien.ac.at (M.J.); 2 Section of Pediatric Trauma Surgery, Department of Trauma Surgery, University Clinic of Orthopedics and Trauma Surgery, Medical University of Vienna, 1090 Vienna, Austria

**Keywords:** open fractures, pediatric trauma, trauma mechanisms, children, adolescents

## Abstract

**Highlights:**

**What are the main findings?**

- Open finger and nasal fractures were the most common injuries.
- Anatomical distribution and injury mechanisms changed markedly with age.
- Craniofacial and confrontation-related injuries predominated in adolescents.

**What are the implications of the main findings?**

- Pediatric open fractures extend well beyond long-bone injuries.
- Diagnostic assessment and treatment should consider age-specific injury patterns.
- Long-bone-focused studies may underestimate the true injury spectrum.

**Abstract:**

**Background:** Open fractures are traditionally associated with high-energy trauma, such as falls and traffic accidents, in healthy adults. However, the epidemiological characteristics of such injuries in children and adolescents remain incompletely defined. Most studies focus on long-bone injuries, while comprehensive epidemiological data across all anatomical regions are limited. This study aimed to analyze and provide an overview of age-dependent patterns of injury mechanisms and anatomical distribution in pediatric open fractures. **Methods:** This retrospective cohort study included all open fractures in patients aged 0–18 years treated at a level-I trauma center between 2002 and 2023. Demographic characteristics, injury mechanisms, anatomical distribution, treatment strategies, and complications were analyzed. **Results:** A total of 814 patients with 849 open fractures were included (mean age 10.5 ± 5.6 years; 67.7% male). Injury mechanisms differed significantly across age groups (*p* < 0.001), shifting from falls in infants and leisure-related injuries in younger children toward sports-related, traffic-related, and confrontation-related injuries in adolescents. Anatomical distribution also varied significantly with age (*p* < 0.001). Upper-extremity fractures predominated in younger children, whereas craniofacial fractures increased with age and became the leading anatomical region among adolescents aged 16–18 years (57.7%). Lower-extremity fractures also increased with age, although they accounted for a smaller proportion of fractures overall (14.4%). Across the entire cohort, open fractures most frequently involved the upper extremity (50.1%), followed by the craniofacial region (35.3%). Finger fractures represented the most common injury location (36.3%), followed by nasal fractures (20.6%). Most fractures were managed conservatively (66.8%), while 33.2% underwent surgical treatment. Complications were documented in 15.2% of patients, with surgical treatment required in 50.0% of these cases; septic complications mainly consisted of pin tract infections associated with external fixation. Persistent sequelae occurred in 4.9%, mainly including range-of-motion limitations and pain. **Conclusion:** Pediatric open fractures showed an age-dependent epidemiological pattern, with upper-extremity injuries predominating in younger children and craniofacial injuries increasing among adolescents. The substantial proportion of craniofacial and distal extremity fractures highlights the importance of considering the full anatomical spectrum of pediatric open fractures beyond long-bone injuries alone.

## 1. Introduction

Open fractures represent a distinct subset of pediatric musculoskeletal trauma. They account for approximately 0.7% to 2% of all fractures in children and adolescents treated in hospital-based pediatric cohorts, although reported incidences vary depending on study design and case definition [1,2].

In children with multiple injuries, the incidence increases to approximately 10% [1,3]. Despite their relatively low frequency, open fractures require particular attention because of associated soft tissue damage and the potential risk of infection [4]. Most established concepts in open fracture management originate from adult trauma populations [5]. In adults, open fractures are typically linked to high-energy mechanisms such as traffic-related accidents and frequently involve long bones of the lower extremity [6]. This high-energy paradigm has shaped classification systems and treatment strategies [7,8].

Pediatric trauma differs from adult trauma in several fundamental aspects. Children have distinct anatomical characteristics, developmental stages, activity profiles, and age-dependent risk exposures [9]. The etiology of injuries in children is multifactorial, with leisure and playground accidents representing common mechanisms in younger children, whereas adolescents are increasingly exposed to organized sports, traffic-related incidents, and interpersonal activities [10,11]. Boys are more frequently affected, and fracture incidence increases with age [9,12]. Developmental factors, including body proportions, maturation of motor skills and cognitive abilities, supervision, mobility, and activity levels, may influence pediatric fracture patterns by modifying both exposure to injury and the biomechanical response to trauma [10,11,13,14]. These age-related differences may therefore contribute to variation in injury mechanisms and anatomical fracture distribution across childhood and adolescence. Most available studies focus on selected anatomical regions, particularly long bones of the extremities [13,14], and suggest that open long-bone fractures are rare. High-energy mechanisms are generally linked to more severe injuries rather than representing the typical presentation in pediatric trauma [15], while sports-related trauma remains a relevant contributing mechanism [16]. Although individual publications have separately addressed selected non-long-bone open fractures [17,18], a comprehensive overview of these injuries within the broader pediatric open-fracture spectrum is still lacking [19]. Consequently, age-dependent injury mechanisms and anatomical fracture distribution remain insufficiently characterized when considering the full spectrum of open fractures beyond long-bone injuries, including craniofacial, hand, foot, and other peripheral fractures.

The aim of this study was therefore to provide a comprehensive epidemiological analysis of open fractures in children and adolescents over more than two decades at a level-I trauma center in Central Europe. In contrast to previous studies that primarily focused on long-bone injuries, this study sought to capture the full spectrum of pediatric open fractures, including peripheral extremity injuries as well as craniofacial fractures, with particular emphasis on anatomical distribution, mechanisms of injury, age-dependent patterns, and complications.

## 2. Materials and Methods

### 2.1. Study Design and Patient Selection

This retrospective single-center cohort study was approved by the Ethics Committee of the Medical University of Vienna (EC-Code: 2075/2023) on 26 February 2025 and was conducted in accordance with the Declaration of Helsinki and its latest amendments. All patients aged 0–18 years who sustained an open fracture and were treated at the Department of Trauma Surgery between January 2002 and December 2023 were identified through the institutional electronic database and screened for eligibility.

**Figure 1:**
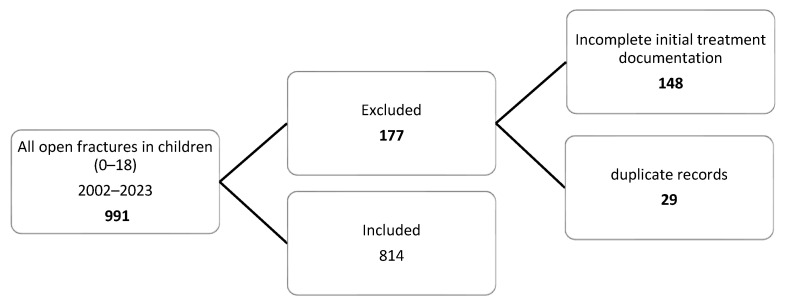
Flowchart of patient selection for pediatric open fractures treated between 2002 and 2023.

Open fractures were defined as fractures associated with disruption of the overlying skin or mucosal barrier, resulting in communication between the fracture site and the external environment, as documented in the medical records. Accordingly, craniofacial fractures, including nasal and facial skull fractures, were classified as open when an associated skin or mucosal wound was reported. Patients were excluded if medical records were missing or if available documentation was insufficient for analysis. Initially, 991 patients were identified. After exclusion of 177 cases, including patients with incomplete initial treatment documentation (*n* = 148) and duplicate records (*n* = 29), 814 patients were included in the final analysis (Figure 1).

### 2.2. Data Collection and Definitions

For all eligible patients, demographic and clinical data were retrospectively extracted from outpatient and inpatient documentation, including operative reports and follow-up records, and entered into a standardized, anonymized database. Collected variables included age, sex, mechanism and circumstances of injury, anatomical fracture location, number of fractures, Gustilo–Anderson classification in open long-bone fractures, polytrauma status, administration and duration of antibiotic therapy, surgical intervention, type of surgical procedure, conservative management, complications, need for revision surgery, and duration of follow-up.

Patients were stratified into predefined age groups reflecting developmental stages and age-specific exposure patterns: infants (0 years), toddlers (1–2 years), preschool children (3–5 years), elementary school children (6–10 years), high school students (11–15 years), and adolescents (16–18 years).

Injury circumstances were categorized according to the documented mechanism or contextual setting as leisure-related incidents, traffic accidents, sports-related injuries, falls, physical confrontations, school-related injuries, work-related accidents, kindergarten-associated injuries, or unknown. Leisure-related incidents comprised non-organized recreational activities and everyday play outside institutional settings, including playground activities, domestic recreational activities, bicycle or scooter use without documented road-traffic involvement, and other non-sport, non-traffic leisure events. Traffic accidents included injuries involving pedestrians, bicycles, scooters, motorcycles, or motor vehicles in a road-traffic context. Sports-related injuries were defined as injuries sustained during organized or clearly identifiable sporting activities. Falls were recorded as a separate mechanism when the fall itself represented the primary injury event rather than being attributable to another documented activity category. For fall-related injuries, low- and high-energy mechanisms were defined according to modified age-adjusted height thresholds: in children younger than 10 years, falls from heights below 2 m were classified as low-energy trauma and falls from 2 m or more as high-energy trauma; in patients aged 10 years or older, the corresponding threshold was set at 3 m [20].

Polytrauma was defined as an Injury Severity Score (ISS) ≥ 16. The Gustilo–Anderson classification was applied only to open long-bone fractures, defined as fractures involving the humerus, radius and/or ulna, femur, and tibia and/or fibula. Complications were defined as documented adverse clinical events during treatment or follow-up that required additional medical or surgical treatment, prolonged the clinical course, or resulted in persistent functional or structural impairment. Complications included infectious events, wound-healing problems, delayed bone healing or nonunion, malalignment, persistent pain, restricted range of motion, neurological or vascular impairment, soft-tissue sequelae, ocular complications, and any event requiring revision surgery. Persistent sequelae were defined as residual symptoms or functional limitations documented at final follow-up.

Descriptive statistics were used to summarize demographic characteristics, injury mechanisms, anatomical fracture distribution, treatment strategies, and complications. Mechanism analyses were performed on a patient basis. Anatomical fracture distribution and treatment patterns according to anatomical region were analyzed on a fracture basis to account for patients with multiple open fractures. Antibiotic administration was assessed at the patient level and descriptively related to the main documented injury region. Complications were recorded on a patient basis. When complications were related to anatomical region or initial treatment category, they were descriptively evaluated using the corresponding fracture-based denominators. Continuous variables are presented as mean ± standard deviation (SD), and categorical variables are presented as absolute numbers and percentages. Age differences between male and female patients were assessed using Welch’s t-test, with Cohen’s d reported as effect size. Associations between categorical variables were analyzed using Pearson’s chi-square test, with Cramer’s V used as effect size. Fisher’s exact test and odds ratios (ORs) with 95% confidence intervals (CIs) were used for selected 2 × 2 comparisons, where appropriate. Age-related trends in individual injury mechanisms and anatomical regions were assessed using binary logistic regression models, with age group entered as an ordinal predictor. A *p*-value < 0.05 was considered statistically significant. Given the exploratory epidemiological design of the study, no adjustment for multiple testing was performed. Statistical analyses as well as the generation of tables and figures were performed using Microsoft^®^ Excel for macOS (Version 16.42; Microsoft Corp., Redmond, WA, USA) and IBM^®^ SPSS^®^ Statistics (Version 27.0.0; IBM Corp., Armonk, NY, USA).

## 3. Results

A total of 814 patients (mean age 10.5 ± 5.6 years) were included in the analysis. Male patients accounted for the majority of the cohort (n = 551; 67.7%), with a mean age of 11.2 ± 5.5 years compared to 9.0 ± 5.5 years in female patients (n = 263; 32.3%). This age difference was statistically significant (mean difference, 2.2 years; Welch’s t-test, *p* < 0.001; Cohen’s d = 0.40). When stratified into predefined age groups, infants accounted for 8 cases (1.0%), toddlers for 68 (8.3%), preschool-aged children for 137 (16.8%), elementary school children for 175 (21.5%), high school students for 203 (24.9%), and adolescents for 223 cases (27.4%) (Table 1).

### 3.1. Injury Mechanisms and Contextual Settings

Among 814 pediatric patients with open fractures, leisure-related incidents were the most frequent injury circumstance (n = 387; 47.9%), followed by traffic accidents (n = 105; 12.9%) and sports-related injuries (n = 102; 12.4%). In particular, falls accounted for 72 cases (8.8%) and physical confrontations for 66 (8.1%). The frequency of injuries sustained in relation to school activities was observed in 39 patients (4.8%), while work-related accidents were documented in 23 cases (2.8%) and kindergarten-associated incidents were noted in 15 instances (1.8%). The mechanism remained unknown in five cases (0.6%) (Table 2).

Age-stratified analysis demonstrated a significant association between age group and injury mechanism (Pearson’s χ^2^(40) = 442.03, *p* < 0.001, Cramer’s V = 0.330). In infants, all open fractures were caused by falls (8/8; 100%). Leisure-related incidents predominated in toddlers, preschool children, and elementary school children, accounting for 57/68 (83.8%), 87/137 (63.5%), and 96/175 (54.9%) cases, respectively, but showed a significant decreasing trend with increasing age. In contrast, traffic-related, sports-related, physical confrontation-related, and work-related injuries increased significantly across age groups. Among adolescents aged 16–18 years, physical confrontation was the leading mechanism (59/223; 26.5%), followed by leisure-related incidents (51/223; 22.9%), traffic accidents (43/223; 19.3%), and sports-related injuries (31/223; 13.9%).

In highschoolers and adolescents, physical confrontations occurred almost exclusively in male patients (62/66, 93.9%). Boys had therefore significantly higher odds of sustaining confrontation-related injuries than girls (11.3% vs. 1.5%; OR 8.21, 95% CI 2.95–22.82; *p* < 0.001). Fall-related injuries showed a nearly equal sex distribution (38 males vs. 34 females).

### 3.2. Mechanisms in Detail

#### 3.2.1. Leisure

Among the 387 leisure-related injuries, the most common subcategories were domestic play and household-related incidents (112 patients, 28.9%), door-crush or entrapment injuries (86 patients, 22.2%), and playground or outdoor recreational accidents (64 patients, 16.5%). Injuries related to the handling of sharp or blunt objects during play accounted for 52 cases (13.4%), while garden, farmyard, animal-related, and other leisure mechanisms were less frequent. In 15 cases (3.9%), the recreational activity or circumstances were not sufficiently detailed.

Leisure-related injuries were predominantly characterized by peripheral extremity fractures, with open finger fractures accounting for the highest proportion (146/387; 37.7%), followed by nasal bone fractures (78/387; 20.2%) and forearm fractures (42/387; 10.9%).

#### 3.2.2. Traffic

Among the 105 traffic-related injuries, bicycle accidents represented the largest subgroup (34/105; 32.4%), followed by pedestrian accidents (20/105; 19.0%), scooter or e-scooter accidents (14/105; 13.3%), motorcycle or moped accidents (12/105; 11.4%), and car occupant accidents (10/105; 9.5%). Other or insufficiently specified traffic mechanisms accounted for the remaining cases.

Traffic-related injuries were primarily characterized by craniofacial and skull fractures, including nasal bone fractures (21/105; 20.0%), other facial skull fractures (17/105; 16.2%), and skull base fractures (8/105; 7.6%). Lower-extremity injuries represented the second major pattern, particularly lower-leg fractures (24/105; 22.9%) and femoral fractures (9/105; 8.6%).

#### 3.2.3. Sports

Among the 102 sports-related injuries, organized team sports represented the largest subgroup (42/102; 41.2%), followed by recreational ball games and informal sporting activities (18/102; 17.6%), organized individual sports (16/102; 15.7%), and wheeled recreational sports (12/102; 11.8%). Winter or seasonal sports and other or insufficiently specified sports mechanisms accounted for the remaining cases.

Among sports-related open fractures, nasal bone fractures represented the most frequent injury pattern (37/102; 36.3%). Additional craniofacial fractures were less frequent (11/102; 10.8%), followed by open finger fractures (28/102; 27.5%) and forearm fractures (16/102; 15.7%).

#### 3.2.4. Falls

Fall-related injuries showed heterogeneous mechanisms. The most common subcategories were falls from furniture (14/72; 19.4%), falls from climbing frames or other playground equipment (11/72; 15.3%), ground-level falls (10/72; 13.9%), and stair-related falls (9/72; 12.5%). Falls from trees accounted for eight cases (11.1%), while falls from windows or windowsills occurred in five cases (6.9%). Other or insufficiently specified fall mechanisms accounted for the remaining cases.

Fall-related injuries were characterized by a predominance of skull and skull base fractures (26/72; 36.1%). Upper-extremity fractures also represented a frequent injury pattern, particularly open finger fractures (24/72; 33.3%) and supracondylar humeral fractures (8/72; 11.1%).

#### 3.2.5. Confrontation

Among the 66 confrontation-related injuries, direct hand impacts represented the predominant mechanism (34/66; 51.5%), followed by kicks or other blunt assaults (8/66; 12.1%), falls occurring during or after the confrontation (7/66; 10.6%), and assaults involving objects (6/66; 9.1%). Other or insufficiently specified confrontation mechanisms accounted for the remaining cases.

Among patients injured during physical confrontations, nasal bone fractures were the most prevalent injury pattern (32/66; 48.5%), followed by open finger fractures (12/66; 18.2%), other craniofacial fractures (10/66; 15.2%), and metacarpal fractures (3/66; 4.5%).

#### 3.2.6. Others

The remaining 82 patients (10.1%) sustained injuries related to school, work, kindergarten, or unknown mechanisms. Among school-related injuries, the most common subcategories were schoolyard or playground incidents (14/82; 17.1%), physical education or supervised school activities (10/82; 12.2%), and door-, crush-, or object-related injuries (8/82; 9.8%). Work-related accidents were mainly caused by machinery or tool-related injuries, crush or entrapment injuries, and falls or blunt trauma. Kindergarten-associated injuries were predominantly related to playground or play activities and door-, crush-, or object-related mechanisms.

These mechanisms were predominantly characterized by distal extremity fractures. Open finger fractures represented the most frequent injury pattern (49/82; 59.8%), followed by toe fractures (17/82; 20.7%). Collectively, these two injury patterns accounted for 66 of 82 cases (80.5%).

### 3.3. Region of Injury

A total of 849 open fractures were identified in this cohort of 814 patients. Overall, 50.1% of fractures (425/849) involved the upper extremity, 35.3% (300/849) involved the craniofacial region, and 14.4% (122/849) involved the lower extremity. Thoracic and pelvic fractures were rare, with only one case each documented (0.1%) (Table 3). The anatomical distribution differed significantly across age groups (Pearson’s χ^2^(20) = 132.02, *p* < 0.001, Cramer’s V = 0.197).

Age-stratified analysis demonstrated a statistically significant shift in anatomical fracture distribution across childhood and adolescence. Region-specific trend analyses showed a significant age-related decrease in upper-extremity fractures and significant age-related increases in craniofacial and lower-extremity fractures. Upper-extremity fractures predominated in younger children, accounting for 48/72 (66.7%) fractures in toddlers, 97/137 (70.8%) in preschool children, and 118/177 (66.7%) in elementary school children. In contrast, craniofacial fractures increased markedly with age and became the predominant anatomical region in adolescents aged 16–18 years, accounting for 138/239 fractures (57.7%), compared with 61/239 (25.5%) upper-extremity fractures. Lower-extremity fractures also increased with age but remained less frequent overall, accounting for 122/849 fractures (14.4%).

### 3.4. Fracture Distribution in Detail

#### 3.4.1. Craniofacial Region

Among open craniofacial fractures (n = 300; mean age 12.2 ± 5.4 years), nasal bone fractures represented the largest subgroup, accounting for 168 cases (19.8% of all fractures; 56.0% of craniofacial fractures). Other open facial fractures included the mandibular (n = 22; 2.6%), orbital (n = 10; 1.2%), and zygomatic region (n = 2; 0.2%).

Open fractures of the skull and calvarium comprised skull base fractures (n = 29; 3.4%) and frontal bone fractures (n = 27; 3.2%), followed by occipital (n = 23; 2.7%) and parietal fractures (n = 16; 1.9%), while temporal fractures were rare (n = 3; 0.4%).

#### 3.4.2. Upper Extremity

Open fractures of the upper extremity (n = 425; mean age 8.6 ± 5.2 years) accounted for 50.1% of all fractures. Finger fractures were by far the most common upper-extremity injury, accounting for 308 cases (36.3% of all fractures; 72.5% of upper-extremity fractures). Forearm fractures were the second most frequent fracture location, observed in 81 cases (9.5%), followed by injuries of the elbow and supracondylar region (21 cases; 2.5%). Metacarpal (8 cases; 0.9%), humeral (5 cases; 0.6%) and carpal fractures (2 cases; 0.2%) constituted only a small proportion of cases.

#### 3.4.3. Lower Extremity

Open fractures of the lower extremity (n = 122; mean age 12.0 ± 4.9 years) accounted for 14.4% of all fractures. Injuries of the lower leg and ankle region represented the largest subgroup, accounting for 51 cases (6.0% of all fractures; 41.8% of lower-extremity fractures). Toe fractures were the second most frequent fracture location, observed in 39 cases (4.6% of all fractures), followed by femoral fractures (17 cases; 2.0%). Fractures involving the tarsal and metatarsal bones were each documented in 6 cases (0.7%), whereas patella fractures were rare (3 cases; 0.4%).

#### 3.4.4. Gustilo–Anderson Classification

Within this cohort, 175 cases (20.6%) were identified as open long-bone fractures. Type I injuries accounted for 138 cases (78.8%), followed by 26 (14.9%) type II fractures and 11 (6.3%) type III fractures. Within the type III subgroup, IIIA injuries were most prevalent (n = 8), whereas IIIB (n = 1) and IIIC fractures (n = 2) were less common.

### 3.5. Treatment Strategies

A total of 282 of 849 open fractures (33.2%) were treated surgically, whereas 567 fractures (66.8%) were managed conservatively. Operative treatment was more frequently required in extremity fractures (239/547; 43.7%) than in craniofacial fractures (43/300; 14.3%).

Conversely, conservative management was predominantly utilized in less complex injuries, particularly open finger and nasal bone fractures. Of the 308 patients with open finger fractures, 261 (84.7%) were treated non-operatively, and of the 168 patients with open nasal bone fractures, 145 (86.3%) were also treated non-operatively.

#### 3.5.1. Surgical Procedures

The most frequently performed procedures were Kirschner wire fixation (n = 84; 29.8%), plate osteosynthesis (n = 53; 18.8%), external fixation (n = 48; 17.0%) and elastic stable intramedullary nailing (ESIN; n = 41; 14.5%) (Table 4).

Nasal bone reduction was the most common craniofacial procedure and was performed in 23 cases.

Overall, screw fixation was carried out in 16 cases across different anatomical regions. Bone reconstruction procedures and amputations were performed in 8 cases each. A single case of elevation of a depressed open skull fracture was documented.

#### 3.5.2. Conservative Management

Conservative management was applied in 567 of 849 open fractures (66.8%) and primarily consisted of immobilization techniques such as finger splints, bandages, forearm and below-knee casts, as well as soft dressings and taping (Table 5). Specifically, conservative treatment predominated in open finger and nasal bone fractures, with 261 of 308 (84.7%) and 145 of 168 fractures (86.3%).

#### 3.5.3. Antibiotic Therapy

Antibiotic therapy was administered in 636 of 814 patients (78.1%), with a mean duration of 6.7 ± 3.7 days. Perioperative antibiotic prophylaxis was generally administered in surgically treated patients; however, the standard regimen changed during the study period. Before 2010, weight-adjusted ampicillin/sulbactam was generally used, whereas from 2010 onward, cefuroxime was used as routine perioperative single-shot prophylaxis. In cases requiring continued antibiotic therapy, intravenous treatment was followed by oral step-down therapy, most commonly with amoxicillin/clavulanic acid, depending on wound conditions and clinical course. In conservatively managed patients without inpatient admission, oral antibiotic therapy was generally prescribed until the first wound check and subsequently discontinued according to local wound findings.

#### 3.5.4. Temporal Trends in Treatment and Antibiotic Use (2002–2012 vs. 2013–2023)

When stratified by time period, the overall proportion of operative treatment remained broadly stable across the study period, with only minor regional variation. Among upper-extremity fractures, operative management slightly decreased from approximately 38% in 2002–2012 to 34% in 2013–2023, whereas the proportion of conservatively treated injuries increased correspondingly from approximately 62% to 66%. Lower-extremity fractures showed consistently higher rates of operative treatment in both periods, with a modest decrease from approximately 76% to 72%. In contrast, craniofacial and skull fractures were predominantly treated non-operatively in both decades, with operative treatment documented in only a small proportion of cases, approximately 13% in 2002–2012 and 15% in 2013–2023.

Antibiotic administration increased across all anatomical regions over time. Among upper-extremity fractures, the proportion of patients receiving antibiotics rose from approximately 75% in 2002–2012 to 93% in 2013–2023, while the documented duration of treatment among antibiotic-treated patients remained relatively stable, increasing only slightly from 6.0 ± 2.9 to 6.5 ± 3.2 days. Lower-extremity fractures showed consistently high rates of antibiotic use in both periods, increasing from approximately 87% to 96%, with comparable treatment durations of 8.3 ± 6.6 and 7.5 ± 4.3 days, respectively. The most pronounced temporal change was observed in patients with craniofacial and skull fractures, where antibiotic administration increased from approximately 49% in the earlier period to 85% in the later period, accompanied by a modest increase in documented treatment duration from 5.9 ± 3.2 to 6.9 ± 3.8 days.

### 3.6. Complications and Outcomes

Follow-up duration was heterogeneous across the cohort, with a mean documented follow-up of 3.6 ± 14.2 months. Overall, 690 of 814 patients (84.8%) recovered without documented complications, whereas 124 patients (15.2%) experienced at least one complication during treatment or follow-up. Surgical treatment of the complication itself was required in 62 of these 124 patients (50.0%).

When complications were descriptively related to anatomical fracture region and initial treatment category, complications were documented in 82 of 282 surgically treated fractures (29.1%) and in 42 of 567 conservatively managed fractures (7.4%). Region-specific complication rates were highest after lower-extremity fractures (44/122; 36.1%), followed by craniofacial/skull fractures (34/300; 11.3%) and upper-extremity fractures (46/425; 10.8%).

Among patients with complications, 108 of 124 (87.1%) had received antibiotic therapy. This corresponded to 108 of 636 patients receiving antibiotics (17.0%), compared with 16 of 174 patients without documented antibiotic therapy (9.2%). Complications were most frequent among adolescents aged 16–18 years (44/223; 19.7%) and high school students aged 11–15 years (36/203; 17.7%).

With respect to injury mechanism, traffic-related injuries showed the highest complication rate (52/105; 49.5%), followed by fall-related injuries (18/72; 25.0%), sports-related injuries (13/102; 12.7%), other or unknown mechanisms (7/82; 8.5%), leisure-related injuries (31/387; 8.0%), and physical confrontation-related injuries (3/66; 4.5%).

Persistent sequelae were documented in 40 patients, corresponding to 32.3% of patients with complications and 4.9% of the overall cohort. The most common persistent finding was limited range of motion (n = 17; 13.7% of patients with complications), followed by persistent pain (n = 7; 5.6%) and malalignment (n = 6; 4.8%). Less frequent persistent outcomes included bone-related complaints (n = 3; 2.4%), nerve damage (n = 2; 1.6%), severe soft tissue damage (n = 2; 1.6%), ocular complications (n = 2; 1.6%), and neurological complaints (n = 1; 0.8%).

Septic complications included 16 cases of pin tract infections associated with external fixation. These infections were managed conservatively in 11 cases, whereas five cases required surgical intervention, including early removal of the external fixator or sequestrectomy. Ten patients died during the clinical course; all deaths occurred in the context of severe polytrauma and were not directly attributable to the open fracture itself.

## 4. Discussion

The present study provides a comprehensive overview of pediatric open fractures treated over a 21-year period at a level-I trauma center. A major finding was that, when all anatomical regions were considered, pediatric open fractures exhibited a wide range of injuries, with a predominance of distal upper-extremity and craniofacial injuries. Specifically, open finger fractures constituted the most prevalent subgroup, accounting for 308 of the total 849 fractures. Subsequently, nasal bone fractures accounted for 168 cases. This observation deviates from the findings of prior studies that concentrated on pediatric open fractures. In these studies, tibial and forearm fractures were identified as the most prevalent injury locations [21,22,23], with hand injuries accounting for a mere 10% of cases [23]. The observed discrepancy can be attributed to the constrained inclusion criteria and the narrow focus on fractures, as the definition of “upper extremity” is limited to long-bone injuries of the humerus, radius, and ulna. The extant literature has focused exclusively on long-bone extremity fractures or on surgically treated fractures, thereby distorting the epidemiological context by highlighting the tibial and forearm region and additionally reporting solely distinct severity levels of limb injuries [17,24,25]. A significant distinction of the present study from existing literature is the comprehensive inclusion of all anatomical regions and injury severities, encompassing fingers, toes, and craniofacial injuries, with the objective of ensuring an accurate epidemiological analysis. The present findings, therefore, do not contradict previous reports [1,9,12,25], but rather complement them by demonstrating that the pediatric open-fracture spectrum is wider when analyzed beyond long-bone injuries. The present cohort also demonstrated a clear age-dependent shift regarding the anatomical distribution of open fractures. Upper-extremity injuries predominated in younger children, which coincides with the literature; however, when all body regions were considered in this analysis, craniofacial fractures became increasingly frequent with age and even represented the leading anatomical region among adolescents. This particular finding extends and contributes to the existing body of literature [2,18] on the anatomical distribution of pediatric open fractures by encompassing all body regions. This finding is relevant for a comprehensive epidemiological assessment, as long-bone- or extremity-focused cohorts may not fully capture the substantial contribution of craniofacial injuries in older children and adolescents. The broad anatomical spectrum observed in this cohort, together with the predominance of leisure-, sports-, and confrontation-related mechanisms, suggests that many pediatric open fractures occur in activity-related contexts rather than exclusively in major high-energy trauma settings. As injury energy was not systematically measured across all mechanisms, this interpretation should be considered descriptive and hypothesis-generating.

Furthermore, this comprehensive epidemiological overview highlights a more extensive documentation of injury mechanisms that have otherwise been underrepresented in current long-bone-focused cohorts [12,25,26]. These mechanisms are now becoming evident and are identified as significant contributors to the overall epidemiological burden. The analysis identified leisure-related incidents as the predominant mechanism, overall, thereby reflecting the significance of daily activities, playground accidents, and recreational exposure in this particular population. The analysis indicated that these incidents were predominantly associated with distal upper-extremity injuries, particularly open finger fractures. This more detailed analysis of leisure-related causes and of exact injury region adds further details to the consistent predominance of hand involvement in domestic, recreational, and play-related activities in younger children [2,9].

Mechanisms that became more prevalent with increasing age, including sports-related trauma, traffic accidents, and physical confrontations, were more frequently associated with craniofacial injuries and lower-extremity fractures in traffic-related cases. A clear shift was demonstrated by adolescents toward higher proportions of traffic-related injuries and injuries related to physical confrontation. This finding indicates an age-dependent change in risk exposure and behavioral patterns. This transition, associated with the aging process, is accompanied by an increasing influence of environmental and behavioral factors. This phenomenon is particularly evident in the context of facial fractures [17,18,27,28]. Sports-related injuries also played a substantial role in this cohort, further emphasizing that pediatric open fractures are often linked to activity-related rather than classic high-energy trauma mechanisms [16]. Therefore, the mechanism-specific fracture distribution provides a plausible explanation for the observed age-dependent anatomical shift: younger children more often sustained distal extremity injuries during play and leisure activities, whereas adolescents increasingly presented with craniofacial injuries in the context of sports, traffic exposure, and physical confrontation. These findings suggest that pediatric open fractures show age-dependent epidemiological characteristics shaped by anatomical vulnerability, supervision patterns, mobility, and age-specific social behavior [3,6,29].

The broad range of injuries was also evident in the treatment approaches and outcomes. Treatment decisions were primarily guided by fracture instability, displacement, contamination, neurovascular compromise, and the extent of associated soft tissue injury. The majority of open fractures, particularly those with minor soft tissue damage, were managed conservatively. This approach was particularly evident in stable fractures of the distal extremity and craniofacial region [17,25,30]. Conversely, operative treatment was primarily indicated for displaced, unstable, macroscopically contaminated, and thus more complex fractures. However, this relatively large proportion of conservative treatment in this study should not lead to the assumption that pediatric open fractures are uniformly benign. These findings suggest that pediatric open fractures comprise both frequently encountered minor-appearing injuries and a smaller but clinically important subgroup of severe injuries with substantial morbidity. This observation is underscored by the fact that complications occurred in a significant but not excessive number of patients with more complex injuries requiring surgical intervention, including lower-extremity fractures, traffic-related trauma, and polytrauma cases [4,5,13]. Septic complications were uncommon and primarily constituted pin tract infections associated with external fixation, which were predominantly manageable without significant long-term sequelae [31]. Mortality was observed exclusively in cases of severe polytrauma, and thus, it should be interpreted as part of the overall trauma burden rather than as fracture-specific mortality.

Several limitations should be considered. The retrospective single-center design may have introduced selection, referral, and documentation bias. As this study was conducted at a level-I trauma center, more severe injuries, polytrauma cases, and complex open fractures may be overrepresented, limiting generalizability to the broader pediatric population. In addition, 177 initially identified cases were excluded, mainly because of incomplete initial treatment documentation or duplicate records. Although these exclusions were necessary to ensure data quality, it cannot be determined whether excluded patients differed from the included cohort.

The long inclusion period from 2002 to 2023 allowed the analysis of a large cohort but may have introduced temporal heterogeneity. Changes in hospital information systems, documentation standards, imaging availability, antibiotic protocols, surgical indications, and follow-up practice may have influenced data completeness and comparability over time. Accordingly, temporal comparisons of treatment and antibiotic use should be interpreted descriptively.

Complication and outcome analyses are limited by retrospective documentation and heterogeneous follow-up duration. Although the mean follow-up duration was formally reported, its interpretation is limited by the highly skewed distribution of the data. Many minor injuries, particularly conservatively treated finger or nasal fractures, often required only short-term routine reassessment, whereas complex surgically treated injuries were usually followed for longer periods due to injury severity, treatment complexity, or complications. This explains the relatively large standard deviation of the mean follow-up duration and limits the interpretability of the mean value alone. Furthermore, the heterogeneous follow-up duration may have led to underreporting of late complications or persistent sequelae in patients with short follow-up. Moreover, higher complication rates in surgically treated fractures or in patients receiving antibiotics should be interpreted cautiously, as these treatments were used according to clinical indication and likely reflect greater injury severity, contamination, soft-tissue damage, hospitalization, and treatment complexity rather than treatment-related effects.

Finally, the comprehensive inclusion of craniofacial, distal extremity, and long-bone injuries increases the epidemiological scope of this study but limits comparability with previous long-bone-focused cohorts. The Gustilo–Anderson classification was applied only to open long-bone fractures and was not suitable for craniofacial, finger, or toe fractures. Therefore, this study was designed to provide a descriptive epidemiological overview of the full spectrum of pediatric open fractures rather than to compare injury severity across anatomical regions.

## 5. Conclusions

Pediatric open fractures showed a distinct age-dependent epidemiological pattern in this cohort, with upper-extremity injuries predominating in younger children and craniofacial injuries becoming more frequent in adolescents. The underlying trauma mechanisms varied across age groups, ranging from leisure-related incidents in younger children to sports-related, traffic-related, and confrontation-related injuries in adolescents. The predominance of craniofacial and distal extremity injuries, together with frequent conservative treatment, highlights the importance of considering the full anatomical spectrum of pediatric open fractures beyond long-bone injuries alone.

## Figures and Tables

**Table 1:** Age group and sex distribution of pediatric patients with open fractures.

Age Groups	Total n (%)	Female (n)	Male (n)
Infants (0 years)	8 (1.0%)	3	5
Toddlers (1–2 years)	68 (8.3%)	33	35
Preschool (3–5 years)	137 (16.8%)	53	84
Elementary School (6–10 years)	175 (21.5%)	67	108
High School (11–15 years)	203 (24.9%)	61	142
Adolescents (16–18 years)	223 (27.4%)	46	177
**Total**	**814 (100%)**	**263**	**551**

**Table 2:** Age-stratified distribution of injury mechanisms and contextual injury settings in pediatric patients with open fractures.

Mechanism/Setting	Total (n)	Percent (%)	0 y (n)	1–2 y (n)	3–5 y (n)	6–10 y (n)	11–15 y (n)	16–18 y (n)	OR	95% CI	*p*	Trend
Leisure	387	47.9%	-	57	87	96	96	51	0.6	0.53–0.67	<0.001	decreasing
Traffic	105	12.9%	-	3	7	20	32	43	1.53	1.28–1.84	<0.001	increasing
Sports	102	12.4%	-	-	11	24	36	31	1.35	1.13–1.61	<0.001	increasing
Falls	72	8.8%	8	6	18	17	11	12	0.66	0.55–0.79	<0.001	decreasing
Physical Confrontation	66	8.1%	-	-	-	-	7	59	11.92	5.68–25.05	<0.001	increasing
School	39	4.7%	-	-	-	18	17	4	1.14	0.88–1.47	0.314	increasing
Work	23	2.8%	-	-	-	-	3	20	8.32	2.79–24.79	<0.001	increasing
Kindergarten	15	1.8%	-	2	13	-	-	-	0.4	0.26–0.62	<0.001	decreasing
Unknown	5	0.6%	-	-	1	-	1	3	1.74	0.73–4.13	0.212	increasing
**Total**	**814**	**100%**	**8**	**68**	**137**	**175**	**203**	**223**				

**Table 3:** Anatomical distribution of pediatric open fractures according to age group.

Region	Total (n)	Percent (%)	0 y (n)	1–2 y (n)	3–5 y (n)	6–10 y (n)	11–15 y (n)	16–18 y (n)	OR	95% CI	*p*	Trend
Upper Extremity	425	50.1%	6	48	97	118	95	61	0.58	0.51–0.65	<0.001	decreasing
Craniofacial	300	35.3%	1	20	29	33	79	138	1.61	1.42–1.82	<0.001	increasing
Lower Extremity	122	14.4%	1	4	11	26	41	39	1.27	1.09–1.49	0.003	increasing
Thorax	1	0.1%	-	-	-	-	-	1	-	-	-	
Pelvis	1	0.1%	-	-	-	-	1	-	-	-	-	
**Total**	**849**	**100%**	**8**	**72**	**137**	**177**	**216**	**239**				

**Footnote:** All values are fracture-based. Totals therefore refer to open fractures rather than patients and may exceed the number of included patients.

**Table 4:** Overview of surgical procedures performed in pediatric open fractures.

Surgical Procedures	Total (n)	Percent (%)	Mean Age ± SD
Kirschner Wire Fixation	84	29.8%	10.6 ± 4.9
Plate Osteosynthesis	53	18.8%	13.7 ± 4.3
External Fixation	48	17.0%	11.6 ± 4.4
ESIN	41	14.5%	9.7 ± 3.3
Nasal Bone Reduction	23	8.2%	14 ± 3.5
Screw Fixation	16	5.8%	15.7 ± 1.3
Bone Reconstruction	8	2.8%	12.6 ± 6.2
Amputation	8	2.8%	15.0 ± 3.7
Elevation Depressed Skull	1	0.4%	-
**Total**	282	100%	11.9 ± 4.7

**Table 5:** Operative versus conservative treatment according to anatomical region.

Anatomical Region	Fracture Site	Total n	Operative n (%)	Conservative n (%)
Craniofacial				
(n = 300)	Nasal Bone	168	23 (13.7)	145 (86.3)
	Skull Base	29	-	29 (100.0)
	Frontal	27	-	27 (100.0)
	Occipital	23	1 (4.3)	22 (95.7)
	Mandibular	22	14 (63.6)	8 (36.4)
	Parietal	16	1 (6.3)	15 (93.8)
	Orbital	10	3 (30.0)	7 (70.0)
	Temporal	3	-	3 (100.0)
	Zygomatic	2	1 (50.0)	1 (50.0)
Upper Extremity				
(n = 425)	Finger	308	47 (15.3)	261 (84.7)
	Radius/Forearm	81	72 (88.9)	9 (11.1)
	Elbow/Supracondylar	21	18 (85.7)	3 (14.3)
	Metacarpal	8	5 (62.5)	3 (37.5)
	Humerus	5	5 (100.0)	-
	Carpal	2	1 (50.0)	1 (50.0)
Lower extremity				
(n = 122)	Tibia/Fibula/Ankle	51	47 (92.2)	4 (7.8)
	Toe	39	13 (33.3)	26 (66.7)
	Femur	17	17 (100.0)	-
	Tarsal/Metatarsal	12	10 (83.3)	2 (16.7)
	Patella	3	3 (100.0)	-
Others				
(n = 2)	Thorax	1	-	1 (100.0)
	Pelvis	1	1 (100.0)	-
**Total**		**849**	**282 (33.2)**	**567 (66.8)**

## Data Availability

The datasets generated and/or analyzed in the current study are not publicly available due to data privacy but are available from the corresponding author on reasonable request.
